# Multi-dimensional characterization of apoptosis in the tumor microenvironment and therapeutic relevance in melanoma

**DOI:** 10.1007/s13402-024-00930-0

**Published:** 2024-03-19

**Authors:** Jing Ye, Benliang Wei, Guowei Zhou, Yantao Xu, Yi He, Xiheng Hu, Xiang Chen, Guanxiong Zhang, Hong Liu

**Affiliations:** 1 Department of Dermatology, Xiangya Hospital, Central South University, Changsha, Hunan 410008 China; 2 National Engineering Research Center of Personalized Diagnostic and Therapeutic Technology, Changsha, Hunan 410008 China; 3 Hunan Key Laboratory of Skin Cancer and Psoriasis, Changsha, Hunan 410008 China; 4 Hunan Engineering Research Center of Skin Health and Disease, Changsha, Hunan 410008 China; 5 Xiangya Clinical Research Center for Cancer Immunotherapy, Changsha, Hunan 410008 China; 6 Big Data Institute, Central South University, Changsha, Hunan 410008 China; 7 Furong Laboratory, Changsha, Hunan China; 8 Department of Urology, Xiangya Hospital, Central South University, Changsha, China

**Keywords:** Plasma cells, Apoptosis, Immunotherapy, Drug sensitivity, Tertiary lymphoid structures

## Abstract

**Purpose:**

Melanoma is widely utilized as a prominent model for the development of immunotherapy, thought an inadequate immune response can occur. Moreover, the development of apoptosis-related therapies and combinations with other therapeutic strategies is impeded by the limited understanding of apoptosis’s role within diverse tumor immune microenvironments (TMEs).

**Methods:**

Here, we constructed an apoptosis-related tumor microenvironment signature (ATM) and employ multi-dimensional analysis to understand the roles of apoptosis in tumor microenvironment. We further assessed the clinical applications of ATM in nine independent cohorts, and anticipated the impact of ATM on cellular drug response in cultured cells.

**Results:**

Our ATM model exhibits robust performance in survival prediction in multiple melanoma cohorts. Different ATM groups exhibited distinct molecular signatures and biological processes. The low ATM group exhibited significant enrichment in B cell activation-related pathways. What’s more, plasma cells showed the lowest ATM score, highlighting their role as pivotal contributors in the ATM model. Mechanistically, the analysis of the interplay between plasma cells and other immune cells elucidated their crucial role in orchestrating an effective anti-tumor immune response. Significantly, the ATM signature exhibited associations with therapeutic efficacy of immune checkpoint blockade and the drug sensitivity of various agents, including FDA-approved and clinically utilized drugs targeting the VEGF signaling pathway. Finally, ATM was associated with tertiary lymphoid structures (TLS), exhibiting stronger patient stratification ability compared to classical “hot tumors”.

**Conclusion:**

Our findings indicate that ATM is a prognostic factor and is associated with the immune response and drug sensitivity in melanoma.

**Supplementary Information:**

The online version contains supplementary material available at 10.1007/s13402-024-00930-0.

## Introduction

Cell death, particularly apoptosis, is undoubtedly the cornerstone of numerous anti-cancer therapies, encompassing traditional chemotherapy and radiotherapy as well as advanced targeted therapy and immunotherapy [1]. Emerging evidence has provided insights into the intricate involvement of apoptosis in tumor biology and the tumor microenvironment, influencing cancer initiation and progression [2, 3]. Some researches emphasize the crucial role of apoptotic cells in adaptive immune responses, as they serve as a source of antigens [4]. When cellular apoptosis occurs, dendritic cells, situated in different skin layers, promptly phagocytize apoptotic cells, transporting antigens to activate T and B cells, thereby stimulating B cells to produce immunoglobulins and undergo clonal expansion [4, 5]. Other studies [6–9] have revealed that apoptotic cells possess a dual nature, influencing macrophage polarization towards M2-like reparatory and regenerative states that promote cancer development via diverse pathways. Additionally, apoptotic cells are preferentially engulfed by M1 macrophages, thus suppressing M1-mediated anti-tumor activity [10, 11]. Collectively, these findings unveil the dynamic plasticity of the tumor microenvironment orchestrated by apoptosis.

However, the precise characteristics of the tumor microenvironment associated with apoptosis in melanoma remain poorly understood. This is particularly important because apoptosis is without a doubt the spearhead of many anti-cancer therapies in melanoma. Gaining a comprehensive understanding of the impact of apoptosis on the immune microenvironment of melanoma and its therapeutic implications, particularly in immunotherapy, can provide novel strategies for the treatment and combination therapies for melanoma patients. This could also advance the identification of the patient population that stands to gain the most from such therapies.

Accumulating evidence substantiates that the influence of B cells on tumor prognosis and immunotherapy is multifaceted [12–15] and context-dependent, contingent upon their intricate interactions with other immune cells and factors within the tumor microenvironment (TME). Ultimately, the clinical outcomes are shaped by the composition and balance of these distinct B cell subsets, an equilibrium intricately governed by the TME milieu [16]. The prevailing notion is that B cell differentiation and the formation of long-lived plasma cells from “education” within the GC (germinal centers), a microenvironment characterized by elevated birth and apoptosis rates [17]. Apart from the impact of B cell and plasma cell apoptosis per se, the influence of overall apoptosis levels on these cells within the immune microenvironment remains uncertain.

This study therefore aimed to develop an apoptosis-related tumor microenvironment signature (ATM) in melanoma and reveal a comprehensive depiction of apoptosis-related tumor microenvironment characterations in multiple dimensions including bulk, single-cell transcriptomics and spatial transcriptomics. Specifically, we shed light on the central role played by plasma cells in orchestrating the dynamic alterations related to apoptosis status by interacting with other immune cells. Finally, our findings indicate that ATM is a prognostic factor and is associated with the immune response and drug sensitivity in melanoma, which establish a theoretical foundation for drug combinations and identify potential markers for immunotherapy response.

## Methods

### Data collection and processing

#### Clinical tissue samples collection

Paraffin sections of melanoma patients, classified as responders or non-responders to anti-PD1 treatment, were collected from Xiangya Hospital and Fudan University Shanghai Cancer Center. All tissue samples were obtained in accordance with the informed consent policy. Detailed clinical information can be found in Tables S1 and S6 of the Supporting Information.

#### RNA sequencing and data processing

In the in-house cohort, a total of 66 pre-treatment tumor specimens from melanoma patients undergoing anti-PD1 treatment were subjected to RNA sequencing (RNAseq). Prior to applying any data filtering criteria, RNA-Seq reads underwent adaptor trimming, and data quality was assessed using the FastQC software (https://github.com/s-andrews/FastQC). Subsequently, the reads were mapped to the human reference genome (GRCh38.p12 assembly) using the default parameters of the HISAT2 [18] software. The mapped reads were then assembled into transcripts or genes using the Stringtie [19] software along with the genome annotation file (http://hg38_ucsc.annotated.gtf). To address the biases arising from sequencing depths and gene lengths, the relative abundance of transcripts/genes was measured using normalized metrics, namely TPM (Transcripts per million mapped reads), and log2-transformed. The resulting normalized expression matrix can be found in Table S7.

#### Melanoma datasets collection

mRNA expression and clinical data from skin cutaneous melanoma (SKCM) samples from The Cancer Genome Atlas (TCGA) were downloaded from the TCGA data portal (https://portal.gdc.cancer.gov/) [20]. 4 GEO skin cutaneous melanoma cohorts (GSE19234, GSE22153, GSE54467, GSE65904) without treatment from Gene-Expression Omnibus (GEO) (https://www.ncbi.nlm.nih.gov/geo/) were included for further analysis (detail information; see Table S1).

#### Immunotherapy-associated datasets collection

Multiple datasets with anti-PD-L1/PD1/CTLA4 cohort were collected in the study to investigate the association between ATM and immunotherapy efficacy and prognosis. The Riaz N cohort [21] (GSE91061: Anti-PD1-treated advanced melanoma) was obtained from Gene-Expression Omnibus (GEO) (https://www.ncbi.nlm.nih.gov/geo/); The Van Allen, E. M cohort [22] (phs000452.v3: anti-PD1/CTLA4-treated metastatic melanoma) was downloaded from dbGaP database (https://www.ncbi.nlm.nih.gov/gap/). Balar AV cohort was collected from IMvigor210, a single-arm Phase 2 study investigating atezolizumab in metastatic urothelial carcinoma (mUC) patients [23, 24]. The Braun DA cohort [25]: anti-PD-1-treated advanced clear cell renal cell carcinoma was collected from prospective clinical trials (NCT01668784) (detail information; see Table S1).

#### Melanoma single cell and spatial transcriptome datasets collection

Processed gene expression profiles for melanoma were retrieved from TISCH (http://tisch.comp-genomics.org/) under accession numbers GSE123139 [26] (46,612 immune cells from 25 melanoma patients), and GSE120575 [27] (16,291 immune cells from 48 tumor samples of melanoma patients treated with checkpoint inhibitors). The spatial data of melanoma was obtained from 10x Genomics (https://www.10xgenomics.com/resources/datasets/human-melanoma-if-stained-ffpe-2-standard) (detail information; see Table S1).

#### Drug response related data

The AUC data and the gene expression matrix for cancer cell lines were downloaded from the CTRP (https://portals.broadinstitute.org/ctrp.v2.1/) [28, 29].

### Construct the ATM model and identify its impact on the prognosis

**Fig. 1:**
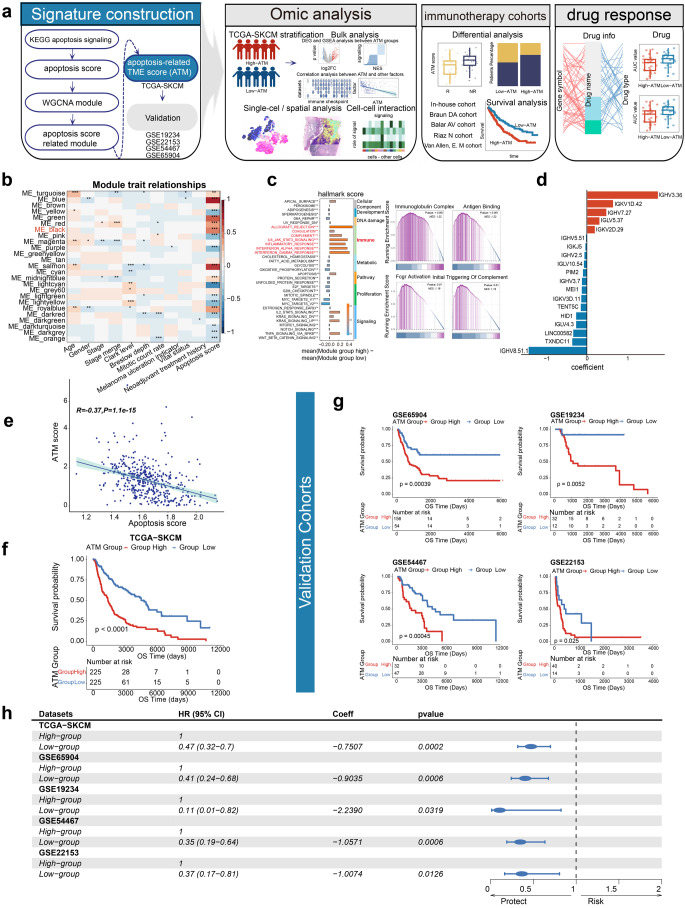
Establishment of the apoptosis-related tumor microenvironment signature (ATM) and its independent prognostic impact in melanoma **a** The overall analytical procedure adopted in our study. **b** The correlation analysis of merged modules and apoptosis score and other clinical features in the TCGA-SKCM cohort. Asterisks denoted *p*-value. **c** The functional enrichment analysis of module genes. The barplot, in the left part, shows the differential hallmark score between module group high and module group low in TCGA-SKCM cohort. The right part shows the GSEA results of module genes. **d** The multicox coefficient of 19 apoptosis-related model genes. Red (positive coefficient) or Blue (negative coefficient). **e** The correlation between ATM score and Apoptosis score. **f** Kaplan–Meier curves for patients with high-ATM and low-ATM scores in the TCGA-SKCM cohort show that patients with low-ATM scores (blue) exhibited better overall survival. **g** Kaplan–Meier curves for patients with high-ATM and low-ATM scores in 4 validation cohorts (GSE65904, GSE19234, GSE54467, GSE22153) show that patients with low-ATM scores (blue) exhibited better overall survival. **h** Forest plot indicates ATM score independently other clinical features influence the prognosis of melanoma patients by the multivariate cox regression analysis in TCGA-SKCM cohort and 4 validation cohorts (GSE65904, GSE19234, GSE54467, GSE22153). Asterisks denoted *p*-value. (“*”*p* < 0.05; “**”*p* < 0.01; “***”*p* < 0.001; “****”*p* < 0.0001; ns was the abbreviation of no significance)

We calculated the Apoptosis score by a single-sample gene set enrichment analysis (ssGSEA) [30] and validated our apoptosis score using experimental data from four distinct datasets with well-documented apoptotic states (GSE196610, GSE147256, GSE137574, GSE242151). Then, weighted gene co-expression network analysis (WGCNA) was used to obtain the apoptosis score associated modules in this study. We analyzed the association between module and immune. Furthermore, we constructed the ATM signature comprising 19 genes through lasso regression, leveraging 216 genes from the apoptosis-related ME_black module. The coefficients of the model genes were optimized using multi-Cox regression (Fig. 1d). Subsequently, the ATM was computed using the predict function of the survival R package [31]. The ATM score, which exhibits a strong correlation with the apoptosis score, was employed for stratifying melanoma patients. Survival analysis and multivariate Cox regression model analysis were performed on the TCGA-SKCM cohort.

### Stratification of tumor samples from TCGA-SKCM cohort and differential analysis, pathways enrichment analysis, and correlation analysis

The TCGA-SKCM samples were divided into two parts by the median cut-off of the ATM score (Table S2). We further compared the differential expression genes between high ATM and low ATM groups in the TCGA-SKCM cohort (Table S3). Significant features were identified by the criterion: mRNA expression |fold change| > 4, FDR < 0.05. These significant features were further subjected to GSEA enrichment analysis (Benjamini and Hochberg correction, FDR < 0.05) by using the fgsea package [32] and the clusterProfiler Package (Table S4) [33]. Then CIBERSORT was used to estimate the differential relative proportions of 22 infiltrating immune cell types between high ATM and low ATM groups based on normalized gene expression data. Furthermore, we evaluated the association between ATM and various clinical features of melanoma, assessing the differences using the Wilcoxon test. The richness value of the T cell receptor/B cell receptor (TCR/BCR) was obtained from Thorsson et al. [34] (https://gdc.cancer.gov/about-data/publications/panimmune).

### Single-cell RNA-seq and spatial RNA-seq analysis of melanoma datasets

We utilized Seurat v3 [35]. R package to pre-process, normalize, and cluster data from two melanoma sample cohorts (GSE120575 and GSE123139) separately. We performed Principal Component Analysis (PCA) on the top 2000 highly variable genes (HVGs) using Seurat v3 [35]. Single-cell ATM scores were calculated based on the model genes and their corresponding coefficients. Uniform Manifold Approximation and Projection (UMAP) embeddings of single-cell RNA-seq profiles enabled the visual representation of annotated cell types and ATM expression. Using subpopulation annotation within the GSE120575 cohort, we extracted B cells and plasma cells for subpopulation annotation. We used ssGSEA [30] to calculate the MHCII signature obtained from Thorsson et al. [34] and compared differential expression across cell types. In addition, we examined the impact of plasma cell ratio on the prognosis and immune response of melanoma patients in the GSE12575 cohort using the Survminer package.“AddModuleScore” function was used to calculate the malignant cell signature score based on the melanoma cell related genes (MLANA, TYR) and the plasma cell signature score based on plasma cell genes (IGHG1, IGHG2) [36].

### Cell-cell interaction analysis of melanoma datasets

To assess the potential cell-cell interactions involving plasma populations and other cell types, we utilized the “CellChat” package, a recently developed tool in R.4.1.2 software [37]. For further analysis of ligand-receptor pairs and individual signaling pathway networks, we selected 10 individual samples characterized by superior survival rates and the highest plasma ratio from the GSE120575 cohort. Default parameterizations were employed throughout the analysis, focusing on Secreted Signaling, ECM-Receptor, and Cell-Cell Contact relationships.

### Identify the impact of the model on prognosis and response to immunotherapy in independent melanoma cohorts

To assess the prognostic and immune response efficacy impact of ATM, we analyzed four melanoma cohorts (GSE19234, GSE22153, GSE54467, GSE65904) comprising untreated samples, an in-house cohort, and four immunotherapy-associated datasets involving anti-PD1 or anti-PD1/CTLA4 treatment. We utilized the Survminer package for analysis and performed multivariate Cox regression model analysis to determine if ATM could function as an independent prognostic factor when considering other confounding factors. Moreover, we compared the proportion of response/non-response patients between high and low ATM groups and the differential ATM values in response and non-response groups using the Wilcoxon test.

### Analysis of drug response in apoptosis status

ATM scores were calculated for each melanoma cell based on gene expression data obtained from the Cancer Therapeutics Response Portal (CTRP). To evaluate the drug response in melanoma cell lines, we examined the Spearman correlation between the area under the curve (AUC) and ATM values of cancer cell lines from CTRP, considering correlations with |Rs| > 0.2 and FDR < 0.05 as statistically significant. A positive Spearman correlation indicated drug resistance, while a negative Spearman correlation indicated drug sensitivity. Specifically, we compared the differential AUC values between the high-ATM and low-ATM groups for apoptosis inducer agents and agents targeting VEGF signaling using the Wilcoxon test.

### Statistical analysis

The Wilcoxon rank sum test was employed to compare the observed differences. The Survminer package was utilized to determine the cutoff point of survival information for each dataset, based on the association between ATM and overall patient survival. To identify the maximum rank statistic and mitigate batch effects, the “surv-cutpoint” function was applied to dichotomize ATM, repeatedly testing all potential cutting points. Subsequently, patient samples were stratified into the high-ATM group and the low-ATM group based on the maximum log-rank statistics. Kaplan-Meier comparative survival analyses were conducted for prognostic analysis, and the log-rank test was employed to ascertain the significance of observed differences. To assess ATM’s independence as a predictor, multivariate Cox regression model analysis was performed, incorporating age, gender, and stage as variables. Spearman correlation was employed to calculate the correlation coefficient between the ATM score and apoptosis score, as well as between ATM and panimmune prognosis factors or the AUC value of drugs (|Rs| ≥ 0.2 and FDR < 0.05 indicating statistical significance). All statistical analyses were two-sided, with P < 0.05 considered statistically significant.

## Results

### Establishment of the apoptosis-related tumor microenvironment signature (ATM) and its independent prognostic impact in melanoma patients

To investigate the characteristics of the tumor microenvironment in patients with distinct overall apoptosis statuses, we calculated the Apoptosis score by a single-sample gene set enrichment analysis (ssGSEA) [30] and validated our apoptosis score using experimental data from four distinct datasets with well-documented apoptotic states (GSE196610, GSE147256, GSE137574, GSE242151) (Fig. S1a). Then, weighted gene co-expression network analysis (WGCNA) was used to obtain the apoptosis score associated modules. We identified a module that exhibited a collective biological function significantly associated with apoptosis score, independent of other clinical characteristics (e.g., age, gender, stage, Clark level, Breslow depth, mitotic count rate, melanoma ulceration indicator, etc.). This selection was based on rigorous analysis of the data, as illustrated in Figs. 1b and S1b. The genes comprising this module were found to be significantly enriched in signaling pathways related to immunoglobulin complexes, antigen binding, Fcgr activation, and the initial triggering of complement, as determined through Geneset Enrichment Analysis (GSEA) (Fig. 1c). Moreover, we observed that the high module score group with a higher apoptosis score exhibited the highest hallmark scores in seven immune-related hallmarks, demonstrating that these module genes reflect immune-related changes in the tumor immune microenvironment (Figs. 1c and S2). Next, we constructed an apoptosis-related model comprising 19 genes using lasso regression, utilizing 216 genes from the apoptosis-related ME_black module. The coefficients of the model genes were optimized using multi-cox regression (Fig. 1d). Subsequently, the apoptosis-related tumor microenvironment signature (ATM) was calculated using the predict function of the survival R package [31]. The ATM score, which exhibited a negative correlation with the apoptosis score (R = -0.37, P = 1.1e-15), was utilized for stratifying melanoma patients (Fig. 1f). The results consistently indicated a negative association between ATM score and apoptosis score across multiple datasets (TCGA-SKCM, GSE19234, GSE22153, GSE54467, GSE65904) (Figs. 1e and S3).

To assess the impact of ATM on the prognosis of melanoma patients, we conducted survival analysis and performed multivariate Cox regression model analysis using the TCGA-SKCM cohort. The results revealed that subgroups with low ATM scores exhibited improved overall survival (OS) and ATM serves as an independent prognostic factor even when considering other potential confounding factors (e.g., age, gender, stage, Clark level, Breslow depth, mutation status) (Figs. 1f and S4a).

To ascertain the prognostic capability of the ATM in melanoma patients, we gathered four independent cohorts (GSE19234, GSE22153, GSE54467, GSE65904) for subsequent survival analysis. The results consistently demonstrated that melanoma patients in the low ATM group exhibited improved overall survival (OS), thus confirming the predictive value of ATM (Fig. 1g). Furthermore, we performed multi-cox analysis on these four datasets, encompassing a total of 389 patients. Remarkably, the results consistently revealed that ATM can serve as an independent prognostic factor for predicting the prognosis of melanoma patients, surpassing other factors such as age, gender, tissue location, mutation status, and stage (Figs. 1h and S4b). The consistent outcomes across multiple independent cohorts strengthen the validity and generalizability of ATM as a reliable predictor of melanoma patient outcomes.

### Plasma cells as pivotal contributors in model interpretation: implications for enhanced prognosis and immune response

**Fig. 2:**
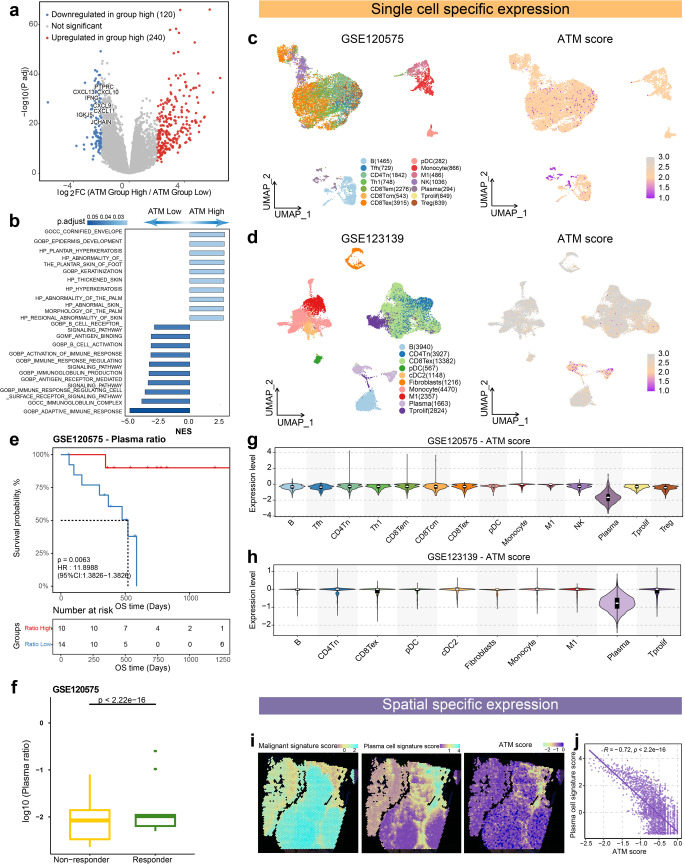
Plasma cells as pivotal contributors in model interpretation: implications for enhanced prognosis and immune response **a** Differential expression genes between the high ATM group and the low ATM group. **b** The top 10 enriched Gene Ontology (GO) signaling pathways associated with differential expression genes between the high ATM group and the low ATM group. **c, d** UMAP plot colored by various cell types or ATM score in GSE120575 and GSE123139 cohorts. **e** Kaplan–Meier curves for patients with high and low plasma ratio in the GSE12575 cohort show that patients with high plasma ratio(red) exhibited better overall survival. **f** The box plot illustrates that immunotherapy responders exhibited a higher plasma ratio in the GSE120575 cohort. **g, h** Violin diagram shows the ATM score across various cell types in GSE120575 and GSE123139 cohorts. **i** Spatial UMAP plot colored by Malignant signature score, plasma cell signature score, and ATM score in 10x Genomics Human Melanoma Spatial Gene Expression Data. **j** The correlation between ATM score and plasma cell signature score in 10x Genomics Human Melanoma Spatial Gene Expression Data

To uncover the molecular characteristics of the TME associated with apoptosis in melanoma patients, we conducted differential expression analysis comparing the high and low ATM groups within the bulk samples of the TCGA-SKCM cohort. This analysis revealed distinct patterns of gene expression, particularly in relation to chemokines such as CXCL13, CXCL11, CXCL10, as well as other factors including PTPRC, JCHAIN, IGKJ5, and IFNG. Notably, these genes exhibited significantly higher expression levels in the low ATM group, indicating a strong potential for antibody secretion within this subgroup (Fig. 2a). Next, we conducted a GSEA analysis to investigate the mRNA-level differences between the high and low ATM groups. Remarkably, the low ATM group exhibited significant enrichment in immune-related signaling pathways. Notably, these pathways included the B cell receptor signaling pathway, B cell activation, immunoglobulin complex, positive regulation for B cell activation, etc. (Figs. 2b and S1d). These findings highlight the pronounced involvement of B cell-related immune pathways in the low ATM group, suggesting a heightened B cell-mediated immune response in this subgroup. Conversely, the high ATM group primarily displayed enrichment in pathways associated with epidermal development, keratinization, keratinocyte differentiation, and skin development (Figs. 2b and S1c). We also analyzed the differential hallmark score between the two ATM score groups in the TCGA-SKCM cohort. We observed that the low ATM score group exhibited significantly higher scores in six immune-related hallmarks, demonstrating that ATM score reflects immune-related changes in the tumor immune microenvironment (Fig. S6). Collectively, these results provide valuable insights into the distinct molecular signatures and biological processes associated with different ATM groups. Besides, to investigate clinical features associated with ATM in melanoma patients, we evaluated ATM across different clinical features, showing that patients with Age < 60, Clark levels I–III and IV had lower ATM relative to those with Clark level V (Fig. S5).

In addition to the insights gained from bulk sample analysis, we extended our investigation to explore the ATM at a single-cell resolution to better understand intra-tumor heterogeneity. Employing graph-based principal component clustering combined with marker-based annotations, we classified cells from the GSE120575 dataset into 14 clusters based on the RNA_snn_res.0.55 and cells from the GSE123139 dataset into 10 clusters based on the RNA_snn_res.0.15, revealing distinct cellular subpopulations (Figs. 2c, d and S7a, b). Notably, plasma cells exhibited the lowest ATM score, further highlighting their potential role as pivotal contributors in our model interpretation (Fig. 2c, d, g, h). Moreover, analyzing the spatial transcriptomic data of Human Skin Melanoma obtained from the official 10X platform consistently demonstrated that plasma cells had the lowest ATM score (Fig. 2i). Interestingly, plasma cells adjacent to tumor cells demonstrates its beneficial spatial location to play its further anti-tumor immunity.

Furthermore, within the GSE120575 cohort, we found that a higher plasma cell ratio was associated with improved survival and enhanced immune response (Fig. 2e, f). Through further analysis, it was determined that this particular cell population predominantly exhibited IgG expression, with a subset displaying IgA (Fig. S7e, f). Moreover, previous investigations corroborated the correlation between IgG+ plasma cells and enhanced survival [38, 39].

### Plasma cells orchestrate a wide spectrum of immune activation state by interacting with cellular constituents of the immune microenvironment

**Fig. 3:**
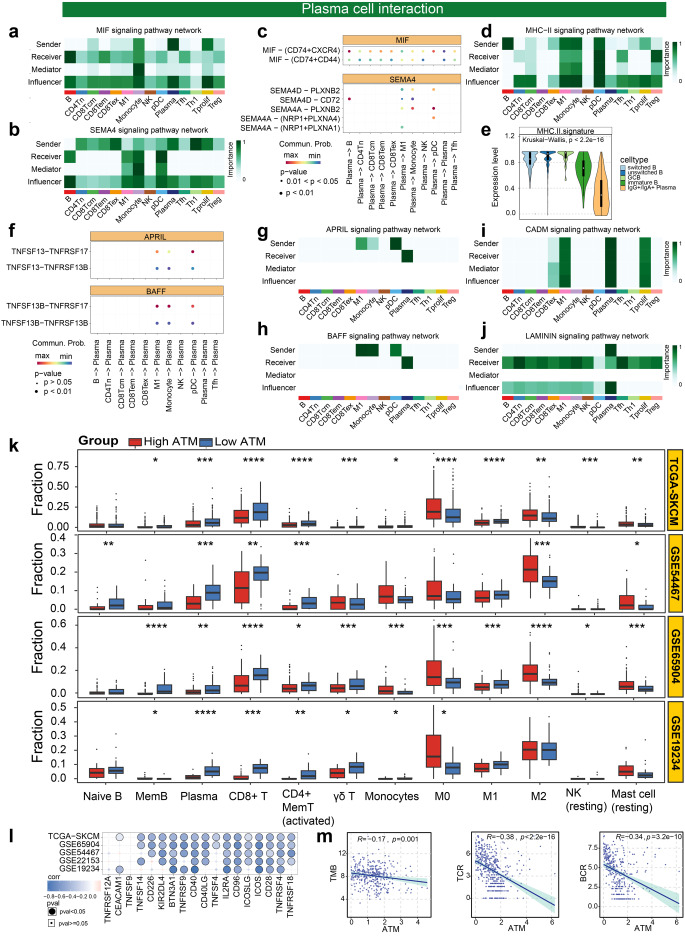
Plasma cells orchestrate a wide immune activation state by interacting with cellular constituents of the immune microenvironment **a, b** Heatmaps showing the relative importance of each cell population based on the computed network centrality measures of MIF signaling pathway network (**a**) and SEMA4 signaling pathway network (**b**) in GSE120575 cohort. **c** The significant ligand-receptor pairs that contribute to the signalings (MIF, SEMA4) sending from plasma to other cell populations. The dot color and size represent the calculated communication probability and *p*-values. *p*-values are computed from one-sided permutation test. **d** Heatmaps showing the relative importance of each cell population based on the computed network centrality measures of MHC-II signaling pathway network in GSE120575 cohort. **e** Violin diagram shows the MHC.II signature expression level across various cell types in GSE120575 B cell subpopulation. **f** The significant ligand-receptor pairs that contribute to the signalings (APRIL, BAFF) sending from other cell populations to plasma cell. **g**–**j** Heatmaps showing the relative importance of each cell population based on the computed network centrality measures of APRIL signaling pathway network (**g**), BAFF signaling pathway network (**h**), CADM signaling pathway network (**i**), and LAMININ signaling pathway network (**j**) in GSE120575 cohort. **k** Bar plot shows the cell types with significantly different cell proportions between two ATM groups which were revealed by the Wilcox test in TCGA-SKCM cohort and 3 validation cohorts (GSE65904, GSE19234, GSE54467). Asterisks denoted *p*-value. **l** Correlation between ATM score and immune checkpoints in TCGA-SKCM cohort and 4 validation cohorts (GSE65904, GSE19234, GSE54467, GSE22153). **m** The correlation between ATM score and TMB, TCR, and BCR in TCGA-SKCM cohort. Asterisks denoted *p*-value. (“*”*p* < 0.05; “**”*p* < 0.01; “***”*p* < 0.001; “****”*p* < 0.0001; ns was the abbreviation of no significance)

Further, cell communication analysis provides the opportunity to study cell-cell interactions based on ligand-receptor binding. Cellchat is thus utilized to conduct an in-depth analysis of the interplay between plasma cells and other cells, elucidating their pivotal role in orchestrating an effective anti-tumor immune response. 10 individual samples exhibiting superior survival rates and the highest plasma ratio from the GSE120575 cohort were taken for an in-depth examination of cellular interactions. The findings unequivocally demonstrated the significant involvement of plasma cells as primary senders and influencers within the MIF signaling pathway network. Remarkably, plasma cells also assume a crucial role as senders and influencers within the SEMA4 signaling pathway network (Fig. 3a, b). It is widely acknowledged that the cytokine macrophage migration inhibitory factor (MIF), a proinflammatory cytokine, plays a key role in inflammatory diseases with chemokine receptors CXCR2, CXCR4, and CD74/CXCR4 complexes as functional receptors. By activating CXCR2, CXCR4, or CD74/CXCR4 complexes, MIF displays chemokine-like functions and acts as a major regulator of inflammatory cell recruitment such as monocytes, T cells, and B cells [40–42]. According to our findings, plasma cells within the MIF signaling pathway exhibit robust chemotactic capabilities towards B cells, pDC and Tfh (Fig. 3a, c), leading to their migration towards the effector site and fostering opportunities for cellular interactions. Moreover, plasma cells in the SEMA4 signaling pathway engage in interactions with B cells, promoting their aggregation through SEMA4D and CD72 ligand receptors [43] (Fig. 3b, c). Notably, our results highlight that B cells play a central role as the primary senders in the MHC-II signaling pathway, facilitating antigen presentation to M1, Monocyte, pDC, Tfh, and Treg (Fig. 3d). We utilize the ssGSEA [30] to estimate the score of MHC.II gene signatures (Fig. 3e) from Thorsson et al. [34]. It is noteworthy that germinal center B cells (GCB) demonstrate the most pronounced MHC-II signature within the B cell subpopulation (Fig. 3e). Several studies have indicated that follicular helper T (Tfh) cells are essential for germinal center formation, affinity maturation, and the development of most high-affinity antibodies and memory B cells [44]. Recent study has also highlighted that pDCs were identified as a component of the T cell zone of TLS, which are major regulators of adaptive antitumor immunity [45]. Collectively, these findings imply that plasma cell chemotaxis facilitates the aggregation of B cells and Tfh which contribute to germinal center formation, allowing GCB to present antigens to Tfh, thereby contributing to subsequent B cell differentiation and the formation of long-lived plasma cells.

Additionally, plasma cell chemotaxis also influences CD8+Tem migration (Fig. 3a, c), thereby enabling CD8+Tem cells to exert their anti-tumor immune function at the effector site. Moreover, plasma cells exhibit chemotactic effects on monocytes (Fig. 3a, c). Our results also indicate that within the APRIL and BAFF signaling pathways, M1, M0, and pDC cells reciprocally interact with plasma cells as senders through the TNFSF13B-TNFRSF17 ligand-receptor interaction (Fig. 3f–h), thereby promoting the longevity of plasma cells [46]. Furthermore, plasma cells, as sender and receiver, played an important role in a series of cell adhesion-related signaling pathways (e.g. CADM, LAMININ) (Fig. 3i, j). Numerous investigations have provided evidence of the involvement of CADM1 (Cell adhesion molecule 1), an immunoglobulin superfamily member, in cell-cell interactions [47]. Furthermore, molecules such as LAMININ, which are part of the ECM-Receptor signaling pathway, offer specific opportunities for interactions between cells and the ECM. These intricate interactions exert direct or indirect control over cellular activities encompassing adhesion, migration, differentiation, and proliferation [48]. Notably, research has highlighted the significance of the adhesion phenotype of plasma cells within the B-cell compartment, contributing to their differentiation and homing [49]. Recent studies have also suggested that chemoattractant cytokines (chemokines), in conjunction with tissue-specific adhesion molecules, coordinate the migration of antibody-secreting cells (ASCs) from lymphoid tissues, where they undergo antigen-driven differentiation, to effector tissues [50]. Taken together, these suggest that this adhesion phenotype of plasma cells is in a “being ready” state for further differentiation or homing to target effector tissues to play anti-tumour immune effects.

These findings highlight the antitumor immune properties of plasma cells themselves, as well as their involvement in coordinating a broad spectrum of immune activation states through interactions with other cells within the immune microenvironment. Consistent results were observed in the immune infiltration analyses of the TCGA-SKCM, GSE54467, GSE65904, and GSE19234 datasets through cibersort [51]. Specifically, the low ATM group exhibited higher proportions of plasma cells, Memory B cells, M1 macrophages, activated CD4+ memory T cells, and CD8+ T cells, indicative of an immunoactivated state. In contrast, the high ATM group displayed higher proportions of M0 macrophages and M2 macrophages, suggesting an immunosuppressive immune microenvironment (Fig. 3k).

In order to investigate the association between ATM and immune checkpoints, we conducted a comprehensive analysis of the correlation between stimulatory immune checkpoint expression and ATM in TCGA-SKCM cohort, as well as four additional GEO cohorts (GSE65904, GSE22153, GSE54467, GSE19234). Remarkably, our findings consistently revealed a negative correlation between ATM and the majority of immune checkpoints (Fig. 3l, Table S5). Notably, the stimulatory immune checkpoints (CD40, IL2RA, CD96, ICOS, CD28, TNFRSF4, BTN3A1) exhibited a consistent negative correlation with ATM across all five datasets. These findings suggest a potential regulatory relationship between ATM and the expression of key immune checkpoints, indicating their coordinated involvement in shaping the tumor immune microenvironment. Subsequently, in order to elucidate the potential mechanism underlying the role of ATM in immunotherapy, we conducted an examination of the association between established immunotherapy biomarkers including tumor mutation burden (TMB), T-cell receptor (TCR), B-cell receptor (BCR), and ATM. We observed the negative correlation between ATM and these biomarkers, indicating a potential interplay between ATM and the immunotherapy response (Fig. 3m).

### ATM serves as a predictor of immunotherapy efficacy

The aforementioned analysis reveals a robust anti-tumor immune response orchestrated by plasma cells in collaboration with other immune cell populations. To investigate the association between ATM and immunotherapy efficacy and prognosis, multiple datasets with anti-PD-L1/PD1/CTLA4 cohorts were collected in the study. The in-house anti-PD1 treatment melanoma patients cohort, two public melanoma patients cohorts with immune checkpoint therapy (the Riaz N cohort [21]/GSE91061: Anti-PD1-treated advanced melanoma (Nivolumab)), and the Van Allen, E. M cohort [22]: Anti–CTLA4-treated metastatic melanoma) and other cohorts with immune checkpoint therapy (the Balar AV cohort [23]/IMvigor210: Anti-PD-L1-treated locally advanced and metastatic urothelial carcinoma and the Braun DA cohort [25]: anti-PD-1-treated advanced clear cell renal cell carcinoma) were taken for analysis.

**Fig. 4:**
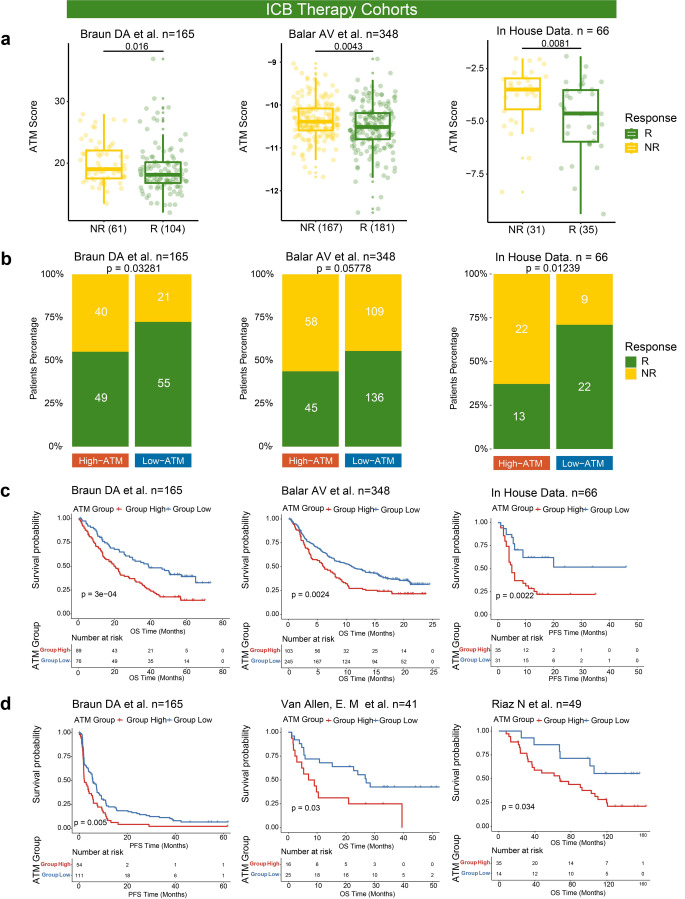
ATM serve as a promising predictor of immunotherapy efficacy **a** Differentiation of ATM in response and non-response groups in two independent immunotherapy cohorts (Braun DA and Balar AV cohorts) and In-house cohort. **b** The proportion of response/non-response patients in high- and low-ATM groups in the Braun DA and the Balar AV cohorts and In-house cohort. **c, d** Kaplan–Meier curves for patients with high ATM and low ATM in four independent immunotherapy cohorts (the Van Allen, E. M; the Balar AV; Braun DA; and the Riaz N cohorts) and In-house cohort show patients with lower ATM (blue) exhibited better overall survival and/or progression-free survival

In Braun DA cohort, Balar AV cohort, and in-house cohort (579 patients), immunotherapy responders were shown to have lower ATM (*p*-value = 0.016 in the Braun DA cohort; *p*-value = 0.0043 in the Balar AV cohort; *p*-value = 0.0081 in the in-house cohort) and the results of the chi-square analysis showed that the proportion of responder would be higher in the low ATM group (*p*-value = 0.033 in the Braun DA cohort; *p*-value = 0.058 in the Balar AV cohort; *p*-value = 0.012 in the in-house cohort) (Fig. 4a, b). Three immunotherapy datasets showed better OS in the group with low ATM, and the Braun DA cohort showed better OS and PFS in the group with low ATM (Fig. 4c, d). All these results exhibited that ATM has a robust independent prognostic ability and predictive power of immunotherapy efficacy.

### Influence of apoptosis-related tumor microenvironment signature (ATM) on anti-cancer drug response

Massive studies have demonstrated that apoptosis can affect drug response mostly in chemotherapy. In order to explore the potential efficacy of influencing other drugs’ sensitivity by apoptosis and develop novel therapeutic hypotheses, we comprehensively depicted the associations between apoptosis-related tumor microenvironment and drug response. We calculated the correlation between the ATM and imputed drug data of drugs in CTRP [28, 29].

**Fig. 5:**
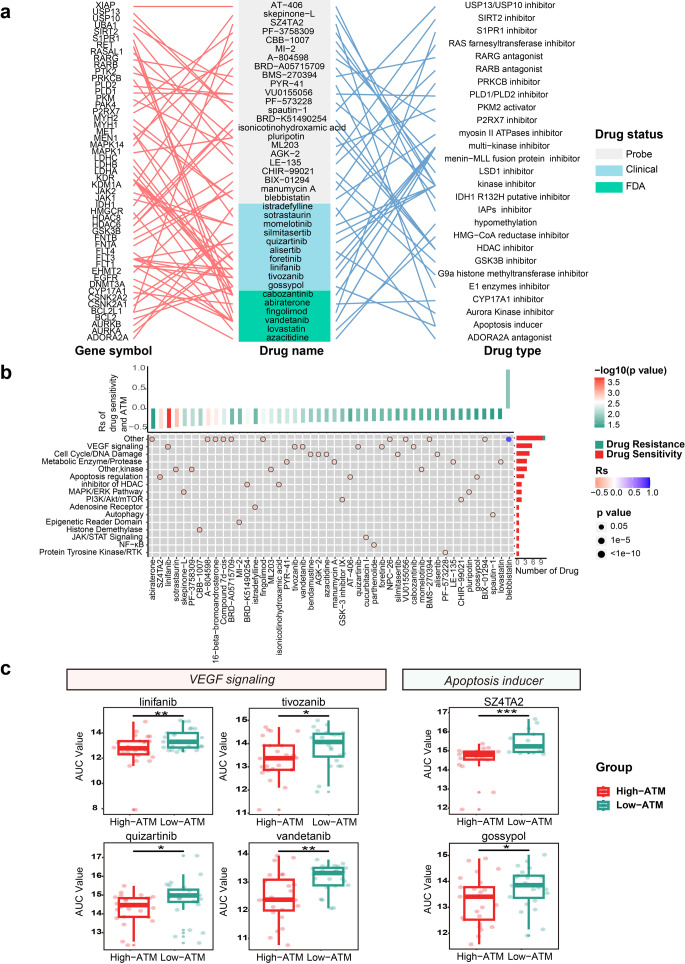
Influence of apoptosis-related tumor microenvironment signature (ATM) on anti-cancer drug response **a** The drug names, targeted gene symbol, and drug type of the three classes of drugs whose drug response correlates with the ATM. **b** Signaling paths targeted by drugs whose drug sensitivity correlates with the ATM. Orange (negative correlation) or blue (positive correlation). **c** Differentiation of AUC value in high- and low-ATM groups in Apoptosis inducer agents (SZ4TA2; gossypol) and agents targeting VEGF signaling (linifanib; tivozanib; quizartinib; vandetanib) was revealed by the Wilcox test. Asterisks denoted *p*-value. (“*”*p* < 0.05; “**”*p* < 0.01; “***”*p* < 0.001; “****”*p* < 0.0001; ns was the abbreviation of no significance)

A total of 51 genes are targeted by 46 drugs including 6 Food and Drug Administration (FDA)-approved drugs, 10 clinically used drugs, and 30 probes that are associated with ATM, most of which are targeted therapy (Fig. 5a). Among these drugs, the area under the curve (AUC value) of 45 drugs is negatively correlated with ATM which means that these drugs are more sensitive in patients with low ATM. CHIR-99021 which is the GSK3B inhibitor is negatively correlated with ATM indicating that it is more sensitive in patients with low ATM with better prognosis (Figs. 4b and 5a). A previous study has also demonstrated that it may function as an antagonist of MYC degradation pathways to transiently elevate MYC levels and then confer chemosensing within a narrow window [52].

Notably, six of these drugs are majoring in the VEGF signally and the AUC of targeting VEGF signaling drugs such as linifanib, tivozanib, quizartinib, and vandetanib are significantly higher in the group with low ATM (Fig. 5b, c). In addition, two apoptosis inducer agents (SZ4TA2, gossypol) are also significantly higher in the group with low ATM (Fig. 5c).

### Apoptosis-related tumor microenvironment signature (ATM) is significantly associated with tertiary lymphoid structures (TLS), exhibiting stronger patient stratification ability compared to classical “hot tumors”

**Fig. 6:**
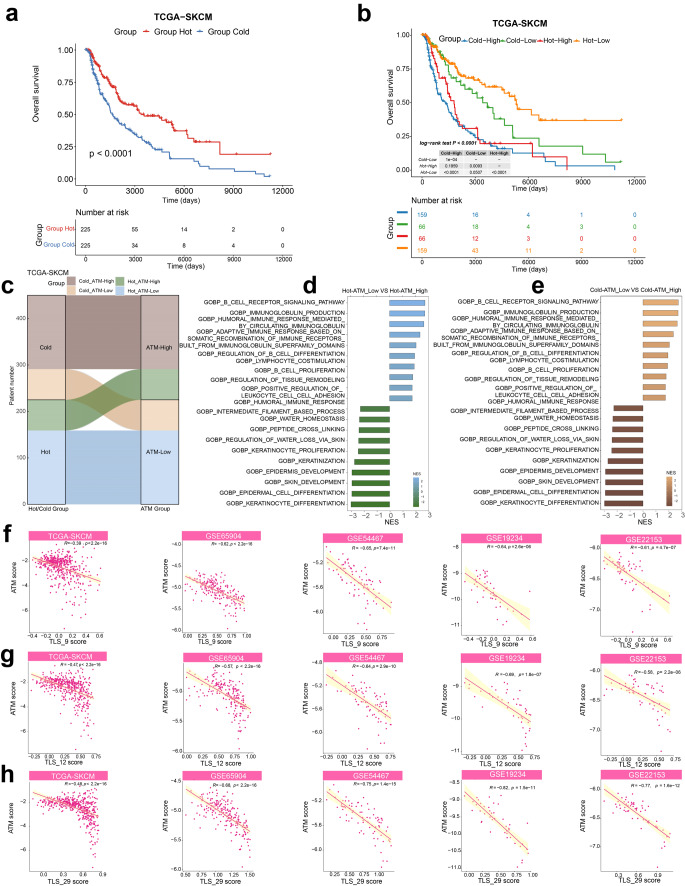
Apoptosis-related tumor microenvironment signature (ATM) is associated with tertiary lymphoid structures (TLS), exhibiting stronger patient stratification ability compared to classical “hot tumors” **a** Kaplan–Meier curves for group hot and cold patients based on the average expression of 12 hot tumor–related genes (CXCL9, CXCL10, CXCL11, CXCR3, CD3, CD4, CD8a, CD8b, CD274, PDCD1, CXCR4, and CCL5) in the TCGA-SKCM cohort show that group hot patients (red) exhibited better overall survival. **b** Kaplan–Meier curves for 4 groups of patients (Cold-High group, Cold-Low group, Hot-High group, and Hot-Low group) based on the average expression of 12 hot tumor and ATM score in the TCGA-SKCM cohort. **c** sankey diagram for four groups of patients stratified by hot tumor score and ATM score in the TCGA-SKCM cohort. **d, e** The top 10 enriched Gene Ontology (GO) signaling pathways associated with differential expression genes between the Hot-ATM low group and Hot-ATM high group (**d**) or between the Cold-ATM low group and Cold-ATM high group (**e**). **f**–**h** The correlation between ATM score and TLS score in TCGA-SKCM cohort and 4 validation cohorts (GSE65904, GSE19234, GSE54467, GSE22153). (ssGSEA was applied to calculated the TLS_9 score (CD79B, CD1D, CCR6, LAT, SKAP1, CETP, EIF1AY, RBP5 and PTGDS); TLS_12 score (CCL2, CCL3, CCL4, CCL5, CCL8, CCL18, CCL19, CCL21, CXCL9, CXCL10, CXCL11 and CXCL13); TLS_29 score (IGHA1, IGHG1, IGHG2, IGHG3, IGHG4, IGHGP, IGHM, IGKC, IGLC1, IGLC2, IGLC3, JCHAIN, CD79A, FCRL5, MZB1, SSR4, XBP1, TRBC2, IL7R, CXCL12, LUM, C1QA, C7, CD52, APOE, PTLP, PTGDS, PIM2, and DERL3 genes)

To explore the association between ATM score and tumor categorization, we compiled genes related to cold and hot tumors from the study by Dong Wang et al., encompassing 12 hot tumor-related genes (CXCL9, CXCL10, CXCL11, CXCR3, CD3, CD4, CD8a, CD8b, CD274, PDCD1, CXCR4, and CCL5), and 3 cold tumor-related genes (CXCL1, CXCL2, and CCL20) [53]. In the TCGA-SKCM cohort, our analysis revealed that the ‘Hot’ group exhibited significantly improved overall survival, aligning with prior research findings. Notably, we further stratified patients based on ATM score and Hot tumor signature. Intriguingly, we observed that irrespective of tumor type (hot or cold), the low ATM group consistently displayed superior survival outcomes compared to the high ATM group. Most importantly, the high ATM score has the capability to identify high-risk patient groups even within ‘hot’ tumors, thus offering crucial insights for patient stratification compared to the conventional ‘hot’ and ‘cold’ tumor classifications. (Fig. 6b, c).

Furthermore, we observed that among patients with either hot or cold tumors, the enriched signaling pathways of the differential gene functional analysis between patients with low ATM scores and those with high ATM scores contained a greater representation of B cell, cell-cell adhesion, tissue remodeling and humoral immune-related pathways (e.g., B cell receptor signaling pathway, humoral immune response mediated circulating immunoglobulin, adaptive immune response based on somatic recombination of immune receptors, regulation of B cell differentiation, lymphocyte costimulation, regulation of tissue remodeling, positive regulation of leukocyte cell-cell adhesion, humoral immune response, etc.) (Fig. 6d, e). This is consistent with the coordinated anti-tumor immune response underscored in our results, focusing on the role of plasma cells. And the observation of plasma cells in a highly adhesive state suggests their readiness for further differentiation or homing to specific effector tissues, enabling them to effectively carry out their anti-tumor immune effects. We further conducted an analysis of the correlation between ATM score and the Tertiary Lymphoid Structure (TLS) signature [54–56], and in all five datasets, we consistently observed that a low ATM score is associated with a higher TLS signature (Fig. 6f–h).

## Discussion

In this study, we elucidate the unclear role of apoptosis in the melanoma microenvironment by establishing an apoptosis-related tumor microenvironment signature (ATM) and investigating its multidimensional alteration features. Our investigation reveals a correlation between ATM and an increased abundance of plasma cells, thereby enhancing the prognostic outlook for patients with melanoma. The chemotactic capability of plasma cells enables the attraction of B cells, Tfh cells, and myeloid cells, consequently fostering their coalescence and facilitating intercellular interactions. As a result, the emergence of long-lasting plasma cells is promoted, establishing an adhesive milieu within the plasma that, in turn, facilitates supplementary plasma cell chemotaxis and the subsequent secretion of antibodies. Notably, plasma cells also exhibit chemotactic tendencies towards CD8+Tem cells, effectively guiding them towards effector sites, where they can exert anti-tumor immune functions. Our study elucidates a comprehensive portrait of apoptosis-associated tumor microenvironment features, highlighting the central role played by plasma cells in orchestrating these dynamic alterations related to apoptosis. Additionally, we discern their potential clinical applications, particularly in the prognostic assessment and predicting immunotherapy responses, accompanied by their insightful implications in evaluating drug sensitivity.

The majority of ATM model genes (13/19) were also observed that they could invole in various biological functions or provided potential prognostic value by previous studies (Table S8). For example, TENT5C was identified as a tumor suppressor, exerting its function by inhibiting Plk4 activity in melanoma [57]. Shilpak et al. discovered that the anti-tumor efficacy of T cell therapy could be significantly enhanced by inhibiting PIM kinase in melanoma mice undergoing adoptive T cell therapy (ACT) [58]. In Hong et al.’s study, a set of 10 genes, including IGKJ5, was constructed to assess the expression levels of TLS, which is important for anti-tumor immune [59]. Previous studies have employed genes such as IGKV1D-42, IGLV5-37, IGKV2D-29, IGHV3-7, IGKV3D-11, and LINC00582 to construct prognostic models for tumors such as melanoma [60–64]. These genes served as pivotal components in our model, and were closely correlated with B/plasma cell functions, underscoring the potential significance of B/plasma cells in anti-tumor immune microenvironment, which was further discussed as below.

An enigmatic facet of the apoptosis program, particularly within the context of the tumor microenvironment, lies in the apoptotic cell’s ability to influence its surrounding tissue milieu beyond mere clearance. Prior investigations have revealed the active involvement of tumor-associated macrophages (TAMs) in the elimination of apoptotic cells, with their accumulation and pro-oncogenic activation intricately linked to tumor growth and angiogenesis. Consequently, the elevated apoptosis levels observed in several cancer types have been implicated in the poorer prognoses of afflicted patients [6–9]. However, in our study, the results show that melanoma patients with low ATM and high apoptosis score exhibit more infiltration of plasma cells. More interestingly, the results of cell-cell communication analysis suggest the adhesion phenotype of plasma cells is in a “being ready” state for further differentiation or homing to target effector tissues to play anti-tumour immune effects. Meanwhile, plasma cells exhibit chemotactic properties and can guide CD8+Tem cells to migrate to the effector site, thereby facilitating their anti-tumor immune functions. Furthermore, the chemotaxis of plasma cells attracts B cells, Tfh cells, and myeloid cells, fostering cellular interactions among them. During the crucial process of antigen presentation by B cells, the chemotactic properties of plasma cells present a unique opportunity for B cells to effectively present antigens to Tfh cells.

Previous investigations have convincingly established that Germinal center B cells (GCBs) seize antigens through the B cell receptor (BCR) and subsequently present processed antigens via MHC complexes to Tfh cells. Enhanced BCR affinity directly corresponds to heightened antigen capture, resulting in a higher density of peptide-MHC complex presentation on the B cell surface [65]. Consequently, this culminates in an amplified T cell help, which intricately governs the selection process. Notably, GC-Tfh cells not only regulate the selection of high-affinity GC B cells but also play a pivotal role in driving the development of long-term humoral immunity by directing GC B cell differentiation into memory B cells and long-lived plasma cells [66–70]. In our findings, we also observed a strong interaction between B cells and GC-Tfh cells in the MHCII signaling pathway, indicating the receipt of assistance from GC-Tfh cells, which significantly contributes to the development of long-term humoral immunity. This partially elucidates why patient groups with low ATM levels demonstrate improved prognoses and enhanced immunotherapy efficacy.

Previous studies have elucidated the crucial role of plasma cells in the tumor microenvironment, primarily exerting their influence through tumor cell killing via opsonization, complement fixation, antibody-dependent cell-mediated cytotoxicity (ADCC), and antibody-mediated phagocytosis. Additionally, plasma cells promote antigen presentation by dendritic cells and drive cytotoxic T cell responses [71–73]. More importantly, our study posits that plasma cells make a significant contribution to the establishment of TLS, thereby creating an immunologically favorable environment. Simultaneously, they collaborate with cytolytic T cells and B cells, enhancing immune activation. Plasma cells demonstrate the capability to recruit B cells, Tfh cells, and myeloid cells by activating CD74/CD44 or CD74/CXCR4 complexes within the MIF signaling pathway. Moreover, plasma cells play a pivotal role in promoting B cell aggregation by mediating the interaction between the SEMA4D-CD72 ligand-receptor pair. This interaction, in turn, facilitates the formation of tumor-associated tertiary lymphoid structures (TLS) and supports essential cellular interactions. These interactions include antigen presentation by B cells, with a specific focus on their role in presenting antigens from germinal center B cells to Tfh cells via the MHC-II complex. This contribution is vital for the maturation and isotype switching of tumor-specific B cells, ultimately culminating in the generation of long-lived plasma cells. Furthermore, plasma cells demonstrate chemotactic properties towards CD8+Tem cells, guiding them to effector sites where they can exert their crucial anti-tumor immune functions. The observation of plasma cells in a highly adhesive state suggests their readiness for further differentiation or homing to specific effector tissues, enabling them to effectively carry out their anti-tumor immune effects. And in all five datasets, we consistently observed that a low ATM score is associated with a higher TLS signature (Fig. 6f–h).

**Fig. 7:**
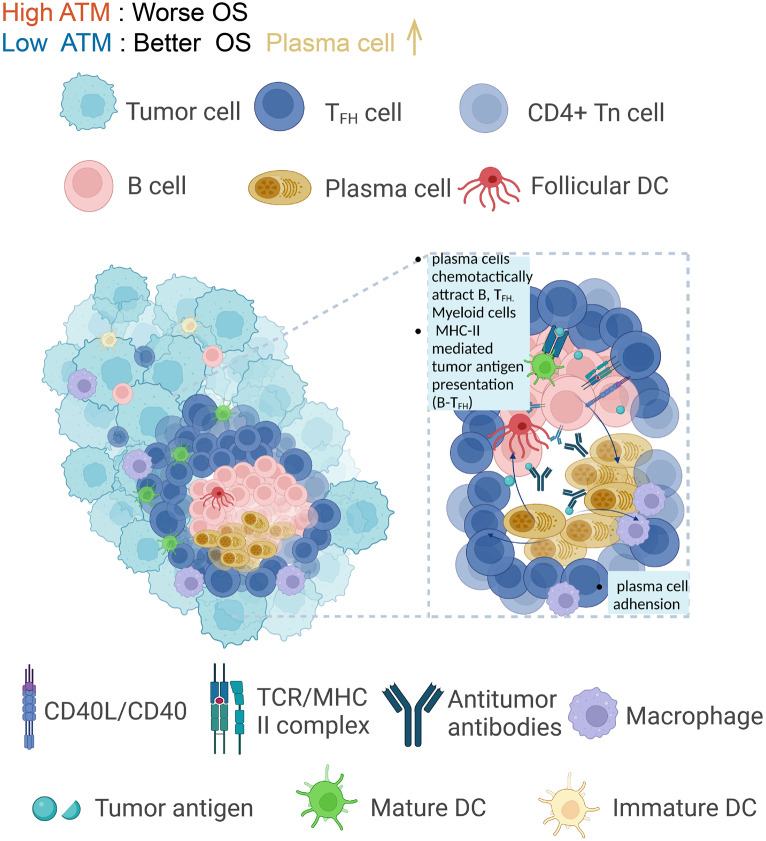
The schematic diagram of plasma cells orchestrating a wide immune activation state by interacting with cellular constituents of the immune microenvironment

Supporting our characterization of plasma cell function, another study on ovarian cancer has revealed consistent findings [74]. Tertiary lymphoid structures (TLS) were frequently observed to be surrounded by dense infiltrates of plasma cells. The presence of plasma cells was associated with the highest levels of CD8+, CD4+, and CD20+ tumor-infiltrating lymphocytes (TILs), as well as the upregulation of numerous cytotoxicity-related gene products. Therefore, we propose that plasma cells contribute to the formation of TLS, creating an immunologically favorable environment, while simultaneously collaborating with cytolytic T cells and B cells to enhance immune activation. This synergistic interplay highlights the cooperative nature of immune responses and underscores the critical role of plasma cells in coordinating anti-tumor immunity (Fig. 7).

Notably, we found that the group with low ATM with relatively higher levels of apoptosis is more sensitive to apoptosis-inducing agents such as SZ4TA2, and gossypol, a clinically used drug. These results suggest a crucial association between both spontaneous and therapeutic agent-induced apoptosis and the mitigation of disease progression. A similar conclusion was observed in another study focused on acute myeloid leukemia (AML) [75]. Previous studies have found that the ‘normalization’ of the tumor vasculature by anti-VEGF agents plays a key role in combinatorial benefits because VEGF inhibition could result in the pruning of endothelial cells not covered by pericytes and a reduction in the tortuosity and hyperpermeability of tumor vessels which are expected to reduce tumor interstitial pressure and lead to enhanced uptake of cytotoxic agents and antibodies by the tumor [76]. This may rationalize our findings that the group with low ATM exhibits heightened sensitivity to VEGF signaling antagonists. Moreover, the low ATM group demonstrates a heightened capacity for secreting high-affinity antibodies, thereby increasing the likelihood of targeting tumor cells while utilizing VEGF signaling antagonists. Anyway, we are dedicated to developing novel therapeutic hypotheses and accelerating the discovery of drugs matched to patients based on their different ATM scores.

### Electronic supplementary material

Below is the link to the electronic supplementary material.

Supplementary Material 1

Supplementary Material 2

Supplementary Material 3

Supplementary Material 4

Supplementary Material 5

Supplementary Material 6

Supplementary Material 7

Supplementary Material 8

Supplementary Material 9

Supplementary Material 10

Supplementary Material 11

Supplementary Material 12

Supplementary Material 13

Supplementary Material 14

Supplementary Material 15

Supplementary Material 16

Supplementary Material 17

## Data Availability

The bulk/single-cell RNA sequencing, spatial transcriptome data, and clinical information of melanoma patients, or patients treated with immune checkpoint blockade were described in the method section “Data collection and processing”. The resources, and tools used in our analyses were described in each method section in the methods.
