# Supplementary figures and images for: Multi-dimensional characterization of apoptosis in the tumor microenvironment and therapeutic relevance in melanoma
Source: Cell Oncol (Dordr). 2024 Mar 19;47(4):1333–53. doi: 10.1007/s13402-024-00930-0 (PMC11322377; doi:10.1007/s13402-024-00930-0)

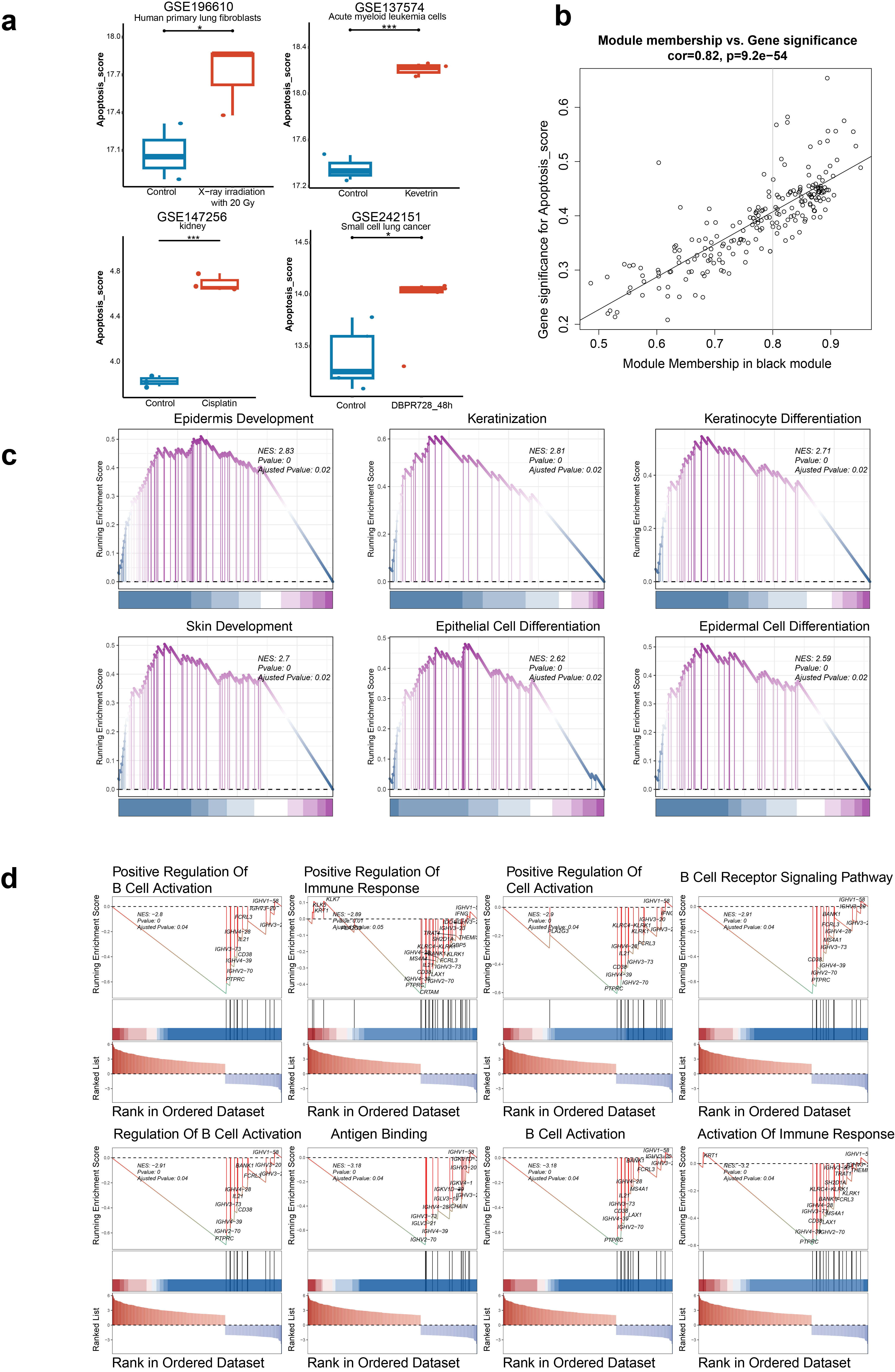

Supplement: Supplementary file 1 — Supplementary Material 1 [file 13402_2024_930_MOESM1_ESM.jpg]

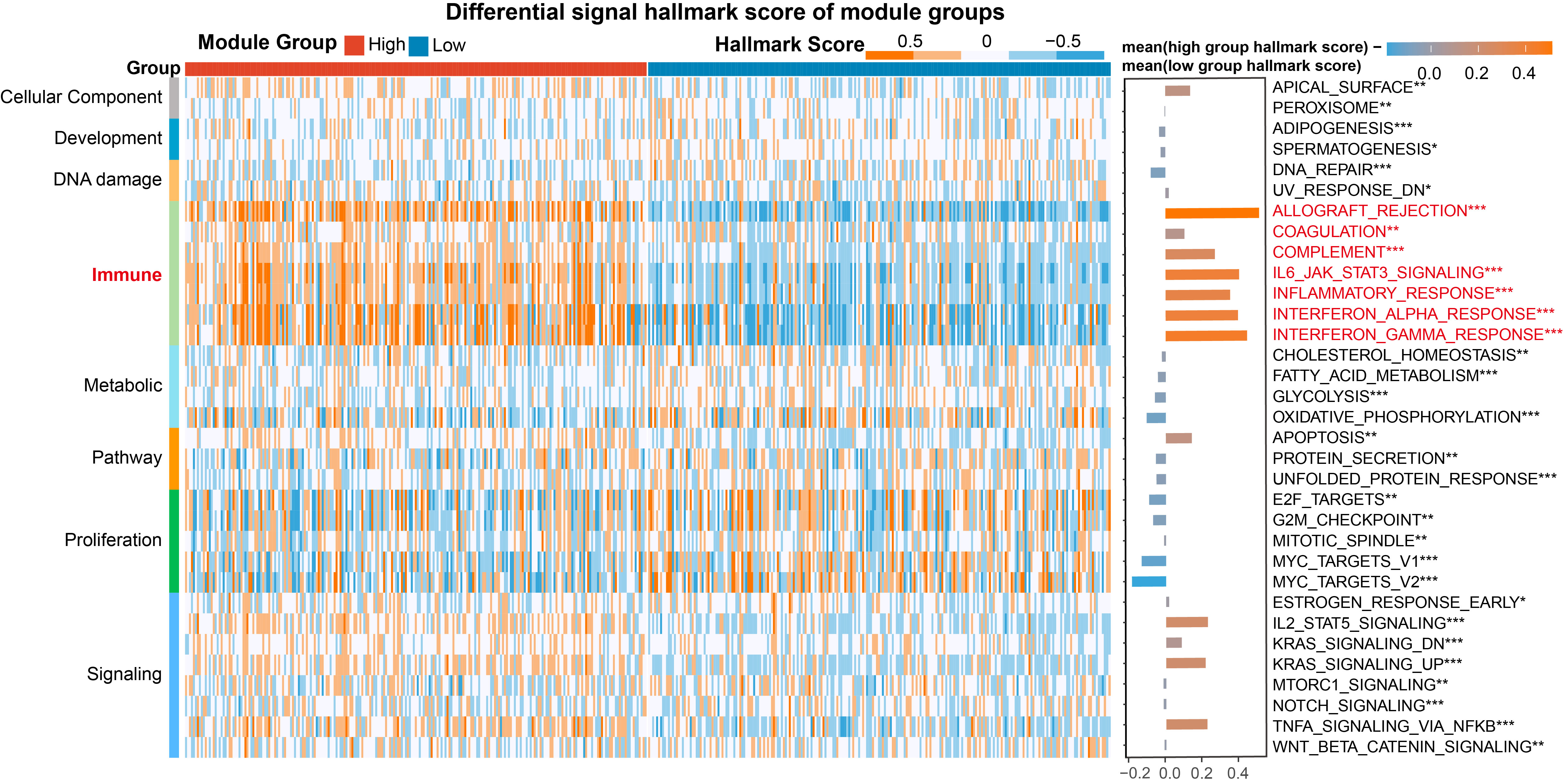

Supplement: Supplementary file 2 — Supplementary Material 2 [file 13402_2024_930_MOESM2_ESM.jpg]

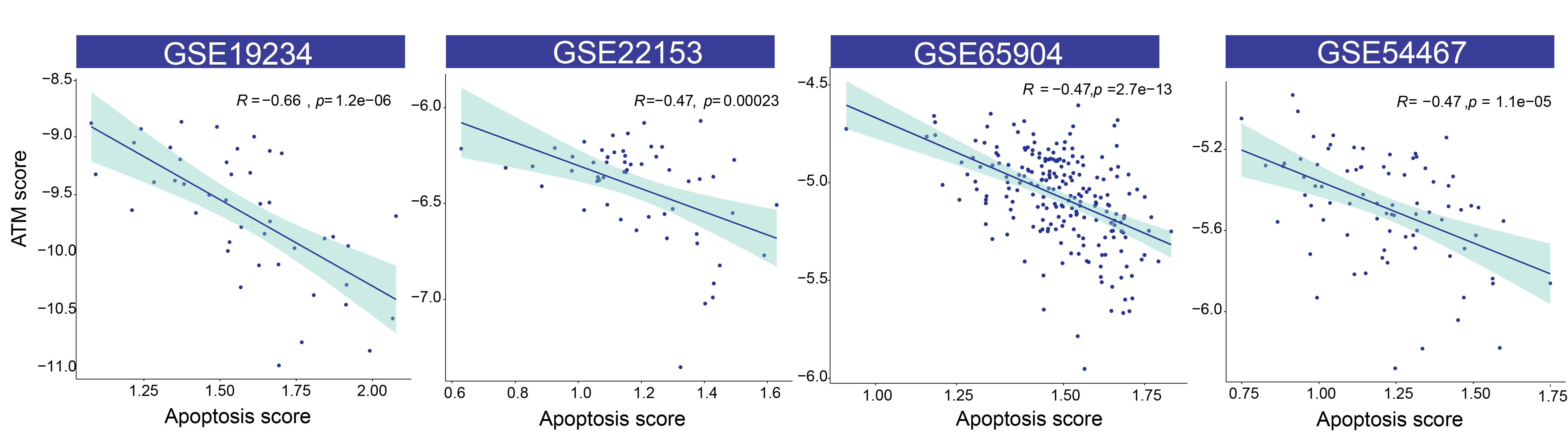

Supplement: Supplementary file 3 — Supplementary Material 3 [file 13402_2024_930_MOESM3_ESM.jpg]

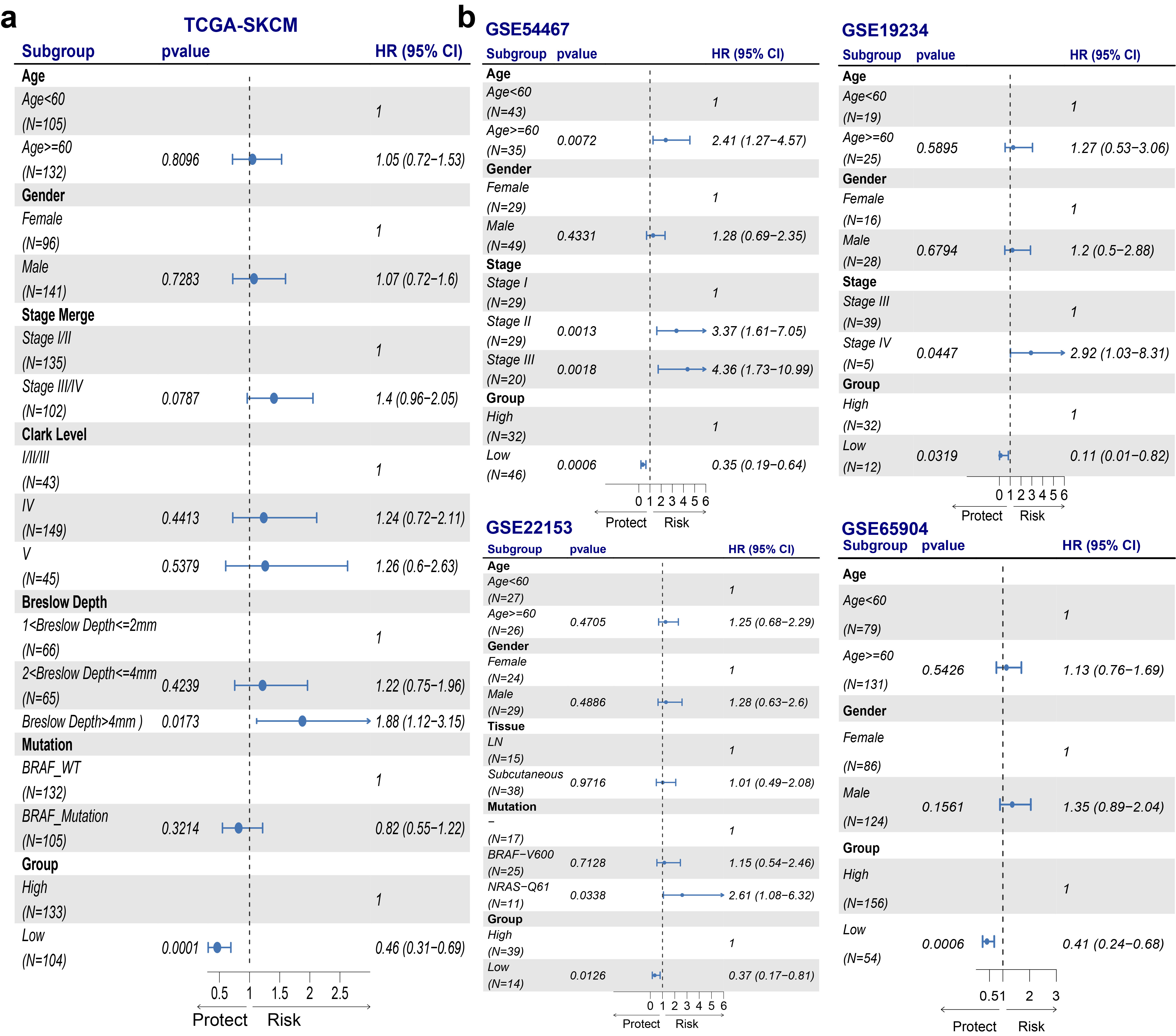

Supplement: Supplementary file 4 — Supplementary Material 4 [file 13402_2024_930_MOESM4_ESM.jpg]

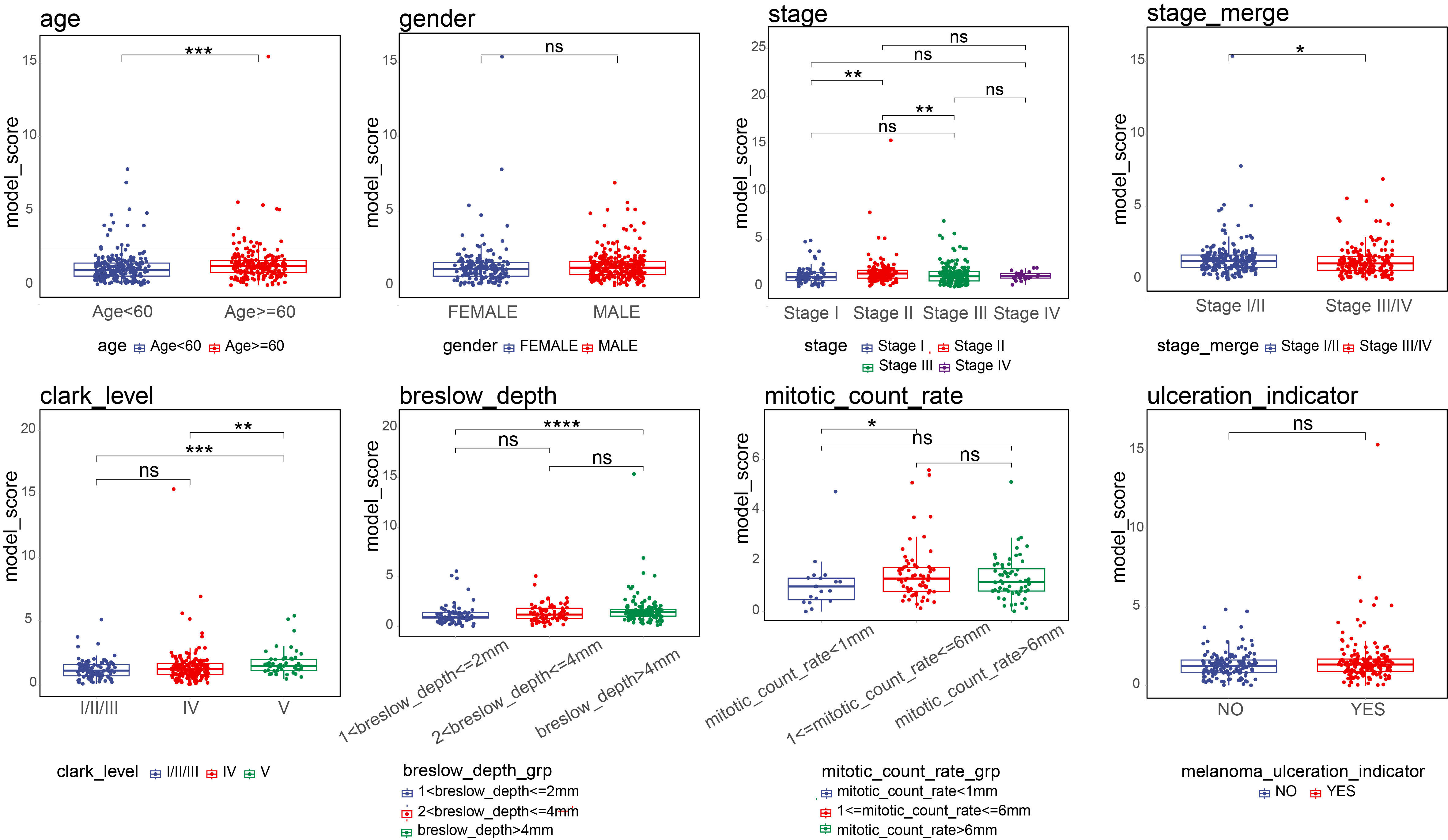

Supplement: Supplementary file 5 — Supplementary Material 5 [file 13402_2024_930_MOESM5_ESM.jpg]

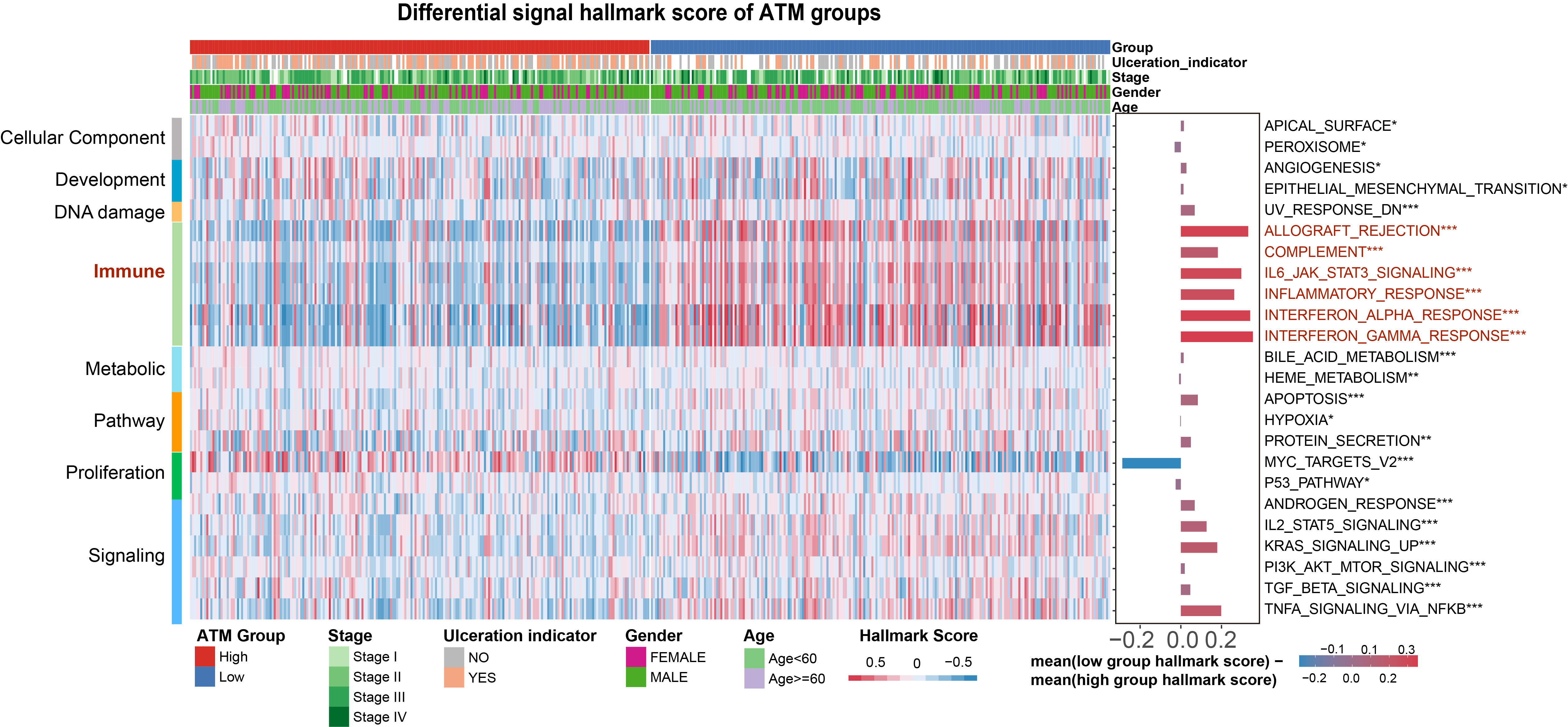

Supplement: Supplementary file 6 — Supplementary Material 6 [file 13402_2024_930_MOESM6_ESM.jpg]

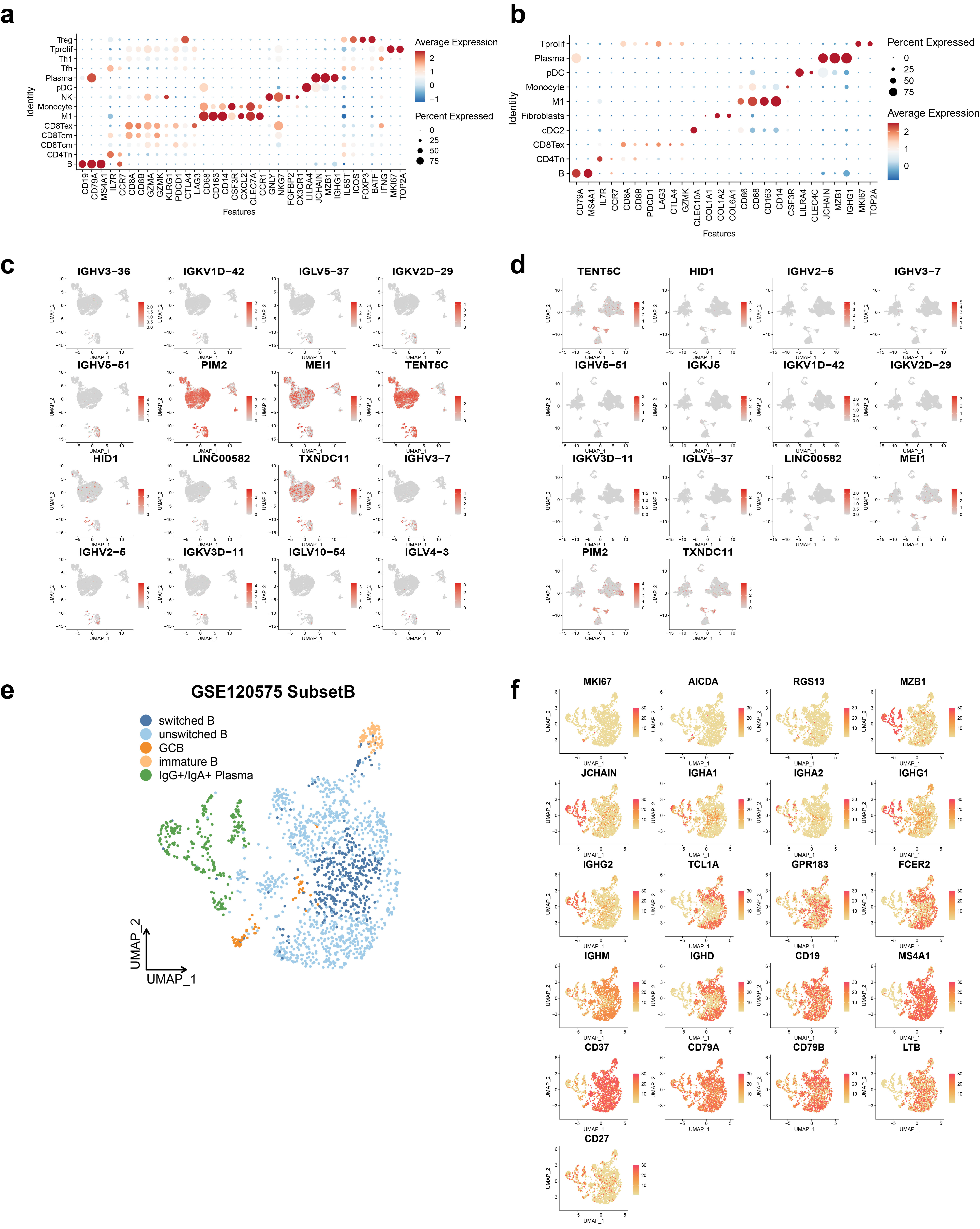

Supplement: Supplementary file 7 — Supplementary Material 7 [file 13402_2024_930_MOESM7_ESM.jpg]
